# Validation of the RightSpot™ device for determination of gastric pH during nasogastric tube placement

**DOI:** 10.1186/1865-1380-6-28

**Published:** 2013-07-17

**Authors:** Charles R Lambert, David Varlotta, Marjorie Posey, Jadie L Heberlein, Janice M Shirley

**Affiliations:** 1 University of Florida College of Medicine, 1600 Archer Road, Gainesville, Florida, 32610, USA; 2 Florida Hospital Tampa Pepin Heart Institute, Dr. Kiran C. Patel Research Institute, 3100 East Fletcher Avenue, Tampa, Florida, 33613, USA

**Keywords:** Nasogastric, pH, Tube, Safety, Placement, RightSpot™

## Abstract

**Background:**

The RightSpotpH™ indicator is a new device designed to facilitate pH-based confirmation of nasogastric tube placement while minimizing exposure of the operator to nasogastric contents. Study objectives: In vivo and in vitro validation of the RightSpotpH™ indicator.

**Findings:**

Patients (23) undergoing general anesthesia had placement of a nasogastric tube and gastric pH electrode catheter following endotracheal intubation. Direct intragastric pH was recorded simultaneously with gastric aspirate pH using the RightSpotpH™ indicator as well as an external pH electrode. In vitro validation of the RightSpotpH™ indicator was performed using standard buffer solutions. Electrode catheter-determined intragastric pH was compared to the RightSpotpH™ indicator result (pH greater or less than 4.5) as was aspirate pH using an external pH electrode. The positive predictive value for the RightSpotpH™ indicator was 0.92 (95% CI 0.62-0.99) and negative predictive value was 1.0 (95% CI 0.48-1.0) when compared to the direct catheter intragastric pH determination. This corresponds to a sensitivity of 1.0 and specificity of 0.83. The positive predictive value for the RightSpotpH™ indicator was 1.0 (95% CI 0.29-0.99) and negative predictive value was 0.75 (95% CI 0.19-0.99) when compared to the external pH electrode determination of aspirate. This corresponds to a sensitivity of 0.91 and specificity of 1.0. Blinded in vitro testing of 46 buffer solution samples of pH 2–7 yielded sensitivity and specificity for correct pH discrimination of 1.0 and 1.0.

**Conclusions:**

The RightSpotpH™ indicator is sensitive and specific for determination of intragastric pH determination as less than or greater than 4.5 as commonly used for nasogastric tube placement.

## Introduction

Placement of nasogastric tubes is one of the most commonly done procedures in the emergency department and critical care settings, and improper placement can lead to significant morbidity. A variety of methods have been utilized to ensure correct placement of nasogastric tubes. These include abdominal radiographs; aspirating gastric contents and measuring pH, bilirubin, pepsin, and trypsin; auscultation; and carbon dioxide measurement, among others [1]. In general, pH determination of the gastric aspirate is considered an acceptable method in clinical practice for nasogastric tube placement and has been endorsed by regulatory agencies [2]. In addition, pH determination (< 5.0) was recently adopted as an evidence-based best practice for determination of correct nasogastric tube placement [3]. This may be done at the bedside by use of pH paper or electrodes; both of these methods involve risk of exposure to the aspirate to the operator during transfer and handling of the fluid.

**Figure 1:**
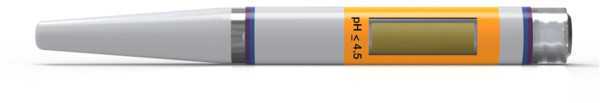
The RightSpotpH™ indicator device connects to a standard nasogastric tube, allowing aspiration of fluid into a chamber containing pH-indicating paper that changes color for pH < 4.5.

The RightSpotpH™ indicator (Figure 1) is a convenient closed system that was developed to minimize operator exposure to gastric aspirate while providing quick and accurate measurement of pH as ≤4.5 or >4.5 using indicator paper technology. The device is placed between the proximal end of the nasogastric tube and a syringe used for aspiration. This study represents the first reported validation of this device in patients as well as in vitro.

## Methods

The Florida Hospital Tampa Institutional Review Board approved this study, which was conducted from January to July 2012. All patients were 21 years of age or older and had a planned elective procedure requiring general anesthesia with endotracheal intubation and placement of a nasogastric tube. A standard nasogastric tube was secured by suture to a pH electrode catheter (Versaflex™) and introduced orally by the attending anesthesiologist following endotracheal intubation. Prior to introduction, the pH catheter was calibrated using buffer solutions per the manufacturer’s directions. Auscultation was also routinely done following tube placement as standard practice.

Following placement, gastric aspirate was withdrawn using a standard syringe attached to the RightSpotpH™ indicator, and a simultaneous reading of direct intragastric pH was taken from the electrode catheter. If enough gastric aspirate was present, pH was also measured with a laboratory pH electrode. Patients were excluded who had grossly bloody nasogastric aspirate. All operators were previously tested for color blindness (Ishihara).

The RightSpotpH™ indicator gives a determination of pH greater or less than 4.5. Results were analyzed comparing the direct intragastric pH versus the RightSpotpH™ indicator reading using Fisher’s exact test. Similarly, the RightSpotpH™ indicator reading was compared to the determination of aspirate pH by the external laboratory pH electrode. An in vitro validation study was done using standard clear buffer solutions of pH 2–7. A total of 46 measurements were made in blinded fashion using the RightSpotpH™ indicator, and data were analyzed in the same manner as for the in vivo comparisons.

## Findings

A total of 23 patients were enrolled in the study. Data were collected from 17, the others having no gastric aspirate (4) or grossly bloody aspirate (2). No complications occurred during the study. Contingency analysis of RightSpotpH™ indicator pH versus directly measured intragastric pH was highly significant (*P* ≤ 0.001). Sensitivity for RightSpotpH™ indicator determination was 1.0 (95% CI 0.71-1.0) and specificity 0.83 (95% CI 0.36-0.99). Positive predictive value for the RightSpotpH™ indicator was 0.92 (95% CI 0.62-0.99) with negative predictive value 1.0 (95% CI 0.47-1.0).

When compared to aspirate pH measured by external pH electrode and a laboratory pH meter, a significant relationship was seen (*P* ≤ 0.009). The positive predictive value for the RightSpotpH™ indicator was 1.0 (95% CI 0.29-0.99) and negative predictive value was 0.75 (95% CI 0.19-0.99). This corresponds to a sensitivity of 0.91 and specificity of 1.0.

Results for the in vitro validation also revealed a significant relationship between RightSpotpH™ indicator determinations and actual pH of clear buffer solutions (*P* ≤ 0.001) with sensitivity of 1.0 (95% CI 0.88-1.0), specificity of 1.0 (95% CI 0.78-1.0), positive predictive value of 1.0 (95% CI 0.88-1.0), and negative predictive value of 1.0 (95% CI 0.78-1.0).

## Discussion

Correct placement of nasogastric tubes is critical for patient safety, and pH testing offers an evidence-based method to assist in this process [3]. The use of pH testing for placement and monitoring of nasogastric tubes has been well described [1-11]. The RightSpotpH™ indicator offers an inexpensive and convenient method to check pH without exposing medical personnel to gastric aspirate and use of pH paper or external electrodes.

This study validates use of the RightSpotpH™ indicator for determination of intragastric pH as less or greater than 4.5 in patients with high sensitivity, specificity, and predictive value. Excellent agreement between RightSpot™ indicator results and direct intragastric pH, external measurement of aspirate pH by electrode and by using standard buffer solutions was observed. It follows that use of the RightSpotpH™ indicator should serve as an efficient, accurate, and potentially safer method for pH-facilitated tube placement than conventional techniques involving handling of body fluids. Use of the device should increase patient safety wherever nasogastric tubes are inserted including the emergency department, critical care areas, perioperatively, and in the field, among others.

### Limitations of the study

This is a simple validation study using the RightSpotpH™ indicator using indicator paper methodology in comparison to intragastric pH detection by catheter electrode or external pH electrode as reference standards. Previous investigations have shown that electrode catheter-determined pH might not always reflect in vitro pH electrode-determined aspirate pH and that this variability also applies to indicator paper-based determinations [9,10,12-14]. Potential reasons for the observed variability between methods are many but probably involve regional variability of intragastric pH, contact of the electrode with the mucosa, and others.

No gastric aspirate was obtained in four patients. This is most likely due to the fact that these patients had been fasting for 24 h prior to their planned procedures.

This work was supported by a grant from EZ-NG, L.L.C., 5635 N. Scottsdale Road, Scottsdale, AZ 85250.

## Competing interests

No competing interests exist for any of the authors. As indicated, a small grant was made to cover the costs of this study.

## Authors’ contributions

CL was the principle investigator for the study and performed data analysis. JH and MP are clinical research coordinators who were assigned to the study, and DV was a co-investigator. JS was the administrative coordinator for the study and also participated in data analysis. All authors read and approved the final manuscript.
